# Tetra-μ_2_-acetato-κ^8^ *O*:*O*′-bis­[(isoquinoline-κ*N*)copper(II)]

**DOI:** 10.1107/S1600536809025732

**Published:** 2009-07-08

**Authors:** Meng-Jiao Li, Jing-Jing Nie, Duan-Jun Xu

**Affiliations:** a Department of Chemistry, Zhejiang University, People’s Republic of China

## Abstract

In the crystal structure of the title compound, [Cu_2_(CH_3_COO)_4_(C_9_H_7_N)_2_], the Cu^II^ cation is coordinated by four acetate anions and one isoquinoline mol­ecule in a distorted square-pyramidal geometry; the Cu^II^ cation is 0.1681 (6) Å from the basal coordination plane formed by the four O atoms. Each acetate anion bridges two Cu^II^ cations to form the centrosymmetric dinuclear complex. Within the dinuclear mol­ecule, the Cu⋯Cu separation is 2.6459 (4) Å. A parallel arrangement of isoquinoline ligands of adjacent complexes is observed in the crystal structure; the face-to-face distance of 3.610 (10) Å suggests there is no π–π stacking between isoquinoline ring systems.

## Related literature

For general background on the nature of π–π stacking, see: Su & Xu (2004 ▶); Xu *et al.* (2007 ▶). For related isoquinoline complexes, see: Clegg & Straughan (1989 ▶); Ivanikova *et al.* (2006 ▶). For a related quinoline complex, see: Pan & Xu (2004 ▶). For the metal atomic deviation from the basal coordination plane in square-pyramidal coordination geometry, see: Xie & Xu (2005 ▶). For the Cu⋯Cu distance in a polymeric Cu^II^ complex, see: Li *et al.* (2007 ▶).
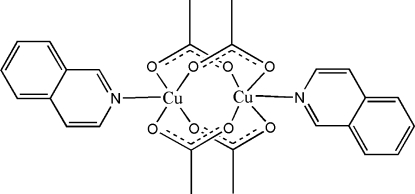


## Experimental

### 

#### Crystal data

[Cu_2_(C_2_H_3_O_2_)_4_(C_9_H_7_N)_2_]
*M* *_r_* = 621.57
Monoclinic, 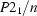

*a* = 12.2278 (3) Å
*b* = 8.1610 (2) Å
*c* = 13.5309 (4) Å
β = 103.827 (8)°
*V* = 1311.13 (7) Å^3^
*Z* = 2
Mo *K*α radiation
μ = 1.67 mm^−1^
*T* = 294 K
0.28 × 0.26 × 0.20 mm

#### Data collection

Rigaku R-AXIS RAPID IP diffractometer
Absorption correction: multi-scan (*ABSCOR*; Higashi, 1995 ▶) *T*_min_ = 0.635, *T*_max_ = 0.720
12480 measured reflections
2997 independent reflections
2638 reflections with *I* > 2σ(*I*)
*R*_int_ = 0.024

#### Refinement

*R*[*F* ^2^ > 2σ(*F* ^2^)] = 0.025
*wR*(*F* ^2^) = 0.074
*S* = 1.06
2997 reflections
172 parameters
H-atom parameters constrained
Δρ_max_ = 0.27 e Å^−3^
Δρ_min_ = −0.40 e Å^−3^

### 

Data collection: *PROCESS-AUTO* (Rigaku, 1998 ▶); cell refinement: *PROCESS-AUTO*; data reduction: *CrystalStructure* (Rigaku/MSC, 2002 ▶); program(s) used to solve structure: *SIR92* (Altomare *et al.*, 1993 ▶); program(s) used to refine structure: *SHELXL97* (Sheldrick, 2008 ▶); molecular graphics: *ORTEP-3 for Windows* (Farrugia, 1997 ▶); software used to prepare material for publication: *WinGX* (Farrugia, 1999 ▶).

## Supplementary Material

Crystal structure: contains datablocks I, global. DOI: 10.1107/S1600536809025732/bq2151sup1.cif

Structure factors: contains datablocks I. DOI: 10.1107/S1600536809025732/bq2151Isup2.hkl

Additional supplementary materials:  crystallographic information; 3D view; checkCIF report

## Figures and Tables

**Table 1:** Selected bond lengths (Å)

Cu—N1	2.1789 (15)
Cu—O1	1.9771 (13)
Cu—O2^i^	1.9728 (13)
Cu—O3	1.9777 (13)
Cu—O4^i^	1.9740 (13)

Symmetry code: (i) 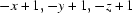.

**Fig. 1.**
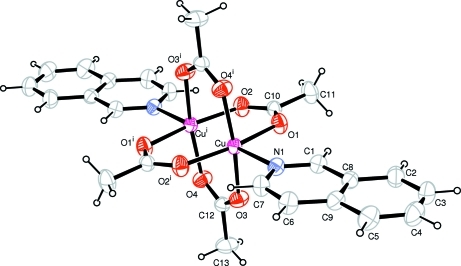
The molecular structure of the title compound with 40% probability displacement (arbitrary spheres for H atoms). The disordered methyl H atoms are not shown for clarify [symmetry code: (i) 1 - x, 1 - y, 1 - z].

**Fig. 2.**
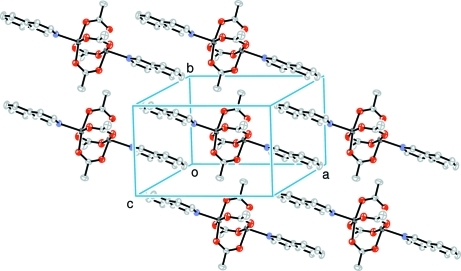
The unit cell packing diagram showing the parallel arrangement of isoquinoline ligands.

## supplementary crystallographic information

### Comment

As part of our ongoing investigation on the nature of π-π stacking (Su & Xu, 2004; Xu *et al.*, 2007), the title complex incorporating isoquinoline ligand has recently been prepared in the laboratory and its crystal structure is reported here.

The molecular structure is shown in Fig. 1. The Cu^II^ cation is coordinated by four O atoms from four acetate anions in the basal plane, an isoquinoline molecule coordinates to the Cu^II^ cation in the apical position to complete the distorted square-pyramidal coordination geometry. The Cu^II^ cation is 0.1681 (6) Å deviated from the basal coordination plane, which is consistent with the situation found in complexes with square-pyramidal coordination geometry (Xie & Xu, 2005). The Cu—N bond in the apical direction is longer than Cu—O bonds in the basal plane by *ca* 0.2 Å, showing the typical Jahn-Teller distortion. Each acetate anion bridges two Cu^II^ cations to form the centro-symmetric dinuclear complex. Within the dinuclear molecule the Cu···Cu separation is 2.6459 (4) Å, similar to 2.642 Å found in a polymeric Cu^II^ complex bridged by thiourea (Li *et al.* 2007).

The parallel arrangement of isoquinoline ligands of adjacent complexes is observed in the crystal structure (Fig. 2). The face-to-face distance of 3.610 (10) Å is close to 3.573 (5) Å found in a quinoline complex (Pan & Xu, 2004) and suggests no π-π stacking between isoquinoline ring systems.

### Experimental

A water-ethanol solution (10 ml, 1:2) of isoquinoline (0.12 ml, 1 mmol) and copper acetate monohydrate (0.10 g, 0.5 mmol) was refluxed for 2.5 h. After cooling to room temperature the solution was filtered. The single crystals of the title compound were obtained from the filtrate after 3 d.

### Refinement

Methyl H atoms were equally disordered over two sites with C—H = 0.96 Å, *U*_iso_(H) = 1.5*U*_eq_(C). Aromatic H atoms were placed in calculated positions with C—H = 0.93 Å and refined in riding mode with *U*_iso_(H) = 1.2*U*_eq_(C).

### Crystal data

**Table:**

[Cu_2_(C_2_H_3_O_2_)_4_(C_9_H_7_N)_2_]	*F*(000) = 636
*M**_r_* = 621.57	*D*_x_ = 1.574 Mg m^−^^3^
Monoclinic, *P*2_1_/*n*	Mo *K*α radiation, λ = 0.71073 Å
Hall symbol: -P 2yn	Cell parameters from 10519 reflections
*a* = 12.2278 (3) Å	θ = 3.0–25.5°
*b* = 8.1610 (2) Å	µ = 1.67 mm^−^^1^
*c* = 13.5309 (4) Å	*T* = 294 K
β = 103.827 (8)°	Chunk, blue
*V* = 1311.13 (7) Å^3^	0.28 × 0.26 × 0.20 mm
*Z* = 2	

### Data collection

**Table:**

Rigaku R-AXIS RAPID IP diffractometer	2997 independent reflections
Radiation source: fine-focus sealed tube	2638 reflections with *I* > 2σ(*I*)
graphite	*R*_int_ = 0.024
Detector resolution: 10.0 pixels mm^-1^	θ_max_ = 27.4°, θ_min_ = 3.0°
ω scans	*h* = −15→15
Absorption correction: multi-scan (*ABSCOR*; Higashi, 1995)	*k* = −10→10
*T*_min_ = 0.635, *T*_max_ = 0.720	*l* = −17→17
12480 measured reflections	

### Refinement

**Table:**

Refinement on *F*^2^	Primary atom site location: structure-invariant direct methods
Least-squares matrix: full	Secondary atom site location: difference Fourier map
*R*[*F*^2^ > 2σ(*F*^2^)] = 0.025	Hydrogen site location: inferred from neighbouring sites
*wR*(*F*^2^) = 0.074	H-atom parameters constrained
*S* = 1.06	*w* = 1/[σ^2^(*F*_o_^2^) + (0.0405*P*)^2^ + 0.4143*P*] where *P* = (*F*_o_^2^ + 2*F*_c_^2^)/3
2997 reflections	(Δ/σ)_max_ = 0.001
172 parameters	Δρ_max_ = 0.27 e Å^−^^3^
0 restraints	Δρ_min_ = −0.40 e Å^−^^3^

### Special details

**Table:**

Geometry. All e.s.d.'s (except the e.s.d. in the dihedral angle between two l.s. planes) are estimated using the full covariance matrix. The cell e.s.d.'s are taken into account individually in the estimation of e.s.d.'s in distances, angles and torsion angles; correlations between e.s.d.'s in cell parameters are only used when they are defined by crystal symmetry. An approximate (isotropic) treatment of cell e.s.d.'s is used for estimating e.s.d.'s involving l.s. planes.
Refinement. Refinement of *F*^2^ against ALL reflections. The weighted *R*-factor *wR* and goodness of fit *S* are based on *F*^2^, conventional *R*-factors *R* are based on *F*, with *F* set to zero for negative *F*^2^. The threshold expression of *F*^2^ > σ(*F*^2^) is used only for calculating *R*-factors(gt) *etc*. and is not relevant to the choice of reflections for refinement. *R*-factors based on *F*^2^ are statistically about twice as large as those based on *F*, and *R*- factors based on ALL data will be even larger.

### Fractional atomic coordinates and isotropic or equivalent isotropic displacement parameters (Å^2^)

**Table:**

	*x*	*y*	*z*	*U*_iso_*/*U*_eq_	Occ. (<1)
Cu	0.411018 (16)	0.55776 (2)	0.528203 (14)	0.03074 (9)	
N1	0.26051 (12)	0.65641 (18)	0.56603 (11)	0.0360 (3)	
O1	0.32898 (12)	0.45444 (18)	0.39965 (11)	0.0457 (3)	
O2	0.47861 (11)	0.35876 (18)	0.35131 (10)	0.0453 (3)	
O3	0.43805 (12)	0.75437 (16)	0.45194 (11)	0.0451 (3)	
O4	0.58673 (12)	0.65621 (17)	0.40330 (11)	0.0457 (3)	
C1	0.16249 (15)	0.6461 (2)	0.49949 (14)	0.0378 (4)	
H1	0.1593	0.5883	0.4396	0.045*	
C2	−0.04146 (17)	0.7044 (3)	0.43993 (16)	0.0496 (5)	
H2	−0.0450	0.6483	0.3794	0.060*	
C3	−0.13598 (18)	0.7741 (3)	0.4580 (2)	0.0576 (6)	
H3	−0.2040	0.7653	0.4096	0.069*	
C4	−0.13180 (19)	0.8585 (3)	0.5482 (2)	0.0595 (6)	
H4	−0.1972	0.9052	0.5593	0.071*	
C5	−0.03322 (19)	0.8738 (3)	0.62062 (18)	0.0547 (5)	
H5	−0.0318	0.9312	0.6803	0.066*	
C6	0.17226 (17)	0.8114 (3)	0.67579 (15)	0.0476 (5)	
H6	0.1791	0.8668	0.7370	0.057*	
C7	0.26360 (16)	0.7388 (3)	0.65421 (14)	0.0428 (4)	
H7	0.3320	0.7457	0.7022	0.051*	
C8	0.06221 (15)	0.7172 (2)	0.51336 (14)	0.0364 (4)	
C9	0.06649 (16)	0.8026 (2)	0.60497 (14)	0.0395 (4)	
C10	0.37462 (16)	0.3780 (2)	0.34010 (13)	0.0365 (4)	
C11	0.2983 (2)	0.3021 (3)	0.24755 (17)	0.0588 (6)	
H11A	0.3428	0.2473	0.2081	0.088*	0.50
H11B	0.2540	0.3861	0.2071	0.088*	0.50
H11C	0.2493	0.2244	0.2685	0.088*	0.50
H11D	0.2213	0.3245	0.2477	0.088*	0.50
H11E	0.3101	0.1857	0.2487	0.088*	0.50
H11F	0.3148	0.3474	0.1873	0.088*	0.50
C12	0.51710 (15)	0.7661 (2)	0.40790 (13)	0.0361 (4)	
C13	0.5292 (2)	0.9275 (3)	0.3568 (2)	0.0589 (6)	
H13A	0.4710	1.0012	0.3656	0.088*	0.50
H13B	0.5226	0.9096	0.2855	0.088*	0.50
H13C	0.6015	0.9742	0.3869	0.088*	0.50
H13D	0.5924	0.9221	0.3264	0.088*	0.50
H13E	0.5408	1.0137	0.4065	0.088*	0.50
H13F	0.4619	0.9491	0.3051	0.088*	0.50

### Atomic displacement parameters (Å^2^)

**Table:**

	*U*^11^	*U*^22^	*U*^33^	*U*^12^	*U*^13^	*U*^23^
Cu	0.02788 (12)	0.03427 (13)	0.03091 (13)	0.00155 (8)	0.00869 (8)	−0.00023 (8)
N1	0.0332 (7)	0.0399 (8)	0.0368 (7)	0.0025 (6)	0.0117 (6)	0.0014 (6)
O1	0.0362 (7)	0.0574 (9)	0.0413 (7)	−0.0006 (6)	0.0050 (6)	−0.0134 (6)
O2	0.0393 (7)	0.0568 (9)	0.0388 (7)	0.0013 (6)	0.0074 (5)	−0.0118 (6)
O3	0.0476 (8)	0.0401 (7)	0.0518 (8)	0.0056 (6)	0.0203 (6)	0.0090 (6)
O4	0.0450 (7)	0.0423 (7)	0.0554 (8)	0.0042 (6)	0.0231 (6)	0.0123 (6)
C1	0.0375 (9)	0.0428 (10)	0.0346 (9)	0.0026 (8)	0.0114 (7)	−0.0010 (7)
C2	0.0396 (10)	0.0552 (12)	0.0504 (11)	0.0017 (9)	0.0034 (9)	−0.0025 (9)
C3	0.0337 (10)	0.0629 (14)	0.0717 (15)	0.0037 (10)	0.0040 (10)	0.0051 (12)
C4	0.0389 (11)	0.0697 (15)	0.0745 (15)	0.0144 (11)	0.0224 (11)	0.0079 (12)
C5	0.0492 (12)	0.0660 (14)	0.0549 (12)	0.0133 (11)	0.0242 (10)	−0.0007 (11)
C6	0.0470 (11)	0.0602 (12)	0.0369 (9)	0.0062 (10)	0.0124 (8)	−0.0086 (9)
C7	0.0355 (9)	0.0547 (11)	0.0377 (9)	0.0015 (9)	0.0076 (7)	−0.0045 (8)
C8	0.0336 (9)	0.0371 (9)	0.0396 (9)	0.0004 (7)	0.0112 (7)	0.0056 (7)
C9	0.0376 (9)	0.0432 (10)	0.0408 (9)	0.0048 (8)	0.0152 (8)	0.0041 (8)
C10	0.0397 (9)	0.0368 (9)	0.0304 (8)	−0.0032 (8)	0.0032 (7)	0.0001 (7)
C11	0.0534 (13)	0.0723 (15)	0.0437 (11)	−0.0073 (11)	−0.0021 (9)	−0.0175 (11)
C12	0.0378 (9)	0.0343 (9)	0.0349 (8)	−0.0040 (8)	0.0059 (7)	0.0025 (7)
C13	0.0721 (16)	0.0412 (11)	0.0687 (15)	−0.0020 (10)	0.0271 (13)	0.0150 (10)

### Geometric parameters (Å, °)

**Table:**

Cu—N1	2.1789 (15)	C4—H4	0.9300
Cu—O1	1.9771 (13)	C5—C9	1.412 (3)
Cu—O2^i^	1.9728 (13)	C5—H5	0.9300
Cu—O3	1.9777 (13)	C6—C7	1.356 (3)
Cu—O4^i^	1.9740 (13)	C6—C9	1.416 (3)
Cu—Cu^i^	2.6459 (4)	C6—H6	0.9300
N1—C1	1.318 (2)	C7—H7	0.9300
N1—C7	1.362 (2)	C8—C9	1.412 (3)
O1—C10	1.251 (2)	C10—C11	1.505 (3)
O2—C10	1.254 (2)	C11—H11A	0.9600
O2—Cu^i^	1.9728 (13)	C11—H11B	0.9600
O3—C12	1.255 (2)	C11—H11C	0.9600
O4—C12	1.249 (2)	C11—H11D	0.9600
O4—Cu^i^	1.9740 (13)	C11—H11E	0.9600
C1—C8	1.409 (3)	C11—H11F	0.9600
C1—H1	0.9300	C12—C13	1.510 (3)
C2—C3	1.362 (3)	C13—H13A	0.9600
C2—C8	1.415 (3)	C13—H13B	0.9600
C2—H2	0.9300	C13—H13C	0.9600
C3—C4	1.391 (3)	C13—H13D	0.9600
C3—H3	0.9300	C13—H13E	0.9600
C4—C5	1.366 (3)	C13—H13F	0.9600
			
O2^i^—Cu—O4^i^	89.28 (6)	O1—C10—O2	125.47 (17)
O2^i^—Cu—O1	167.80 (6)	O1—C10—C11	117.25 (18)
O4^i^—Cu—O1	89.03 (6)	O2—C10—C11	117.28 (18)
O2^i^—Cu—O3	89.10 (6)	C10—C11—H11A	109.5
O4^i^—Cu—O3	167.77 (6)	C10—C11—H11B	109.5
O1—Cu—O3	90.00 (6)	H11A—C11—H11B	109.5
O2^i^—Cu—N1	97.34 (6)	C10—C11—H11C	109.5
O4^i^—Cu—N1	97.72 (6)	H11A—C11—H11C	109.5
O1—Cu—N1	94.86 (6)	H11B—C11—H11C	109.5
O3—Cu—N1	94.51 (6)	C10—C11—H11D	109.5
O2^i^—Cu—Cu^i^	85.07 (4)	H11A—C11—H11D	141.1
O4^i^—Cu—Cu^i^	84.27 (4)	H11B—C11—H11D	56.3
O1—Cu—Cu^i^	82.74 (4)	H11C—C11—H11D	56.3
O3—Cu—Cu^i^	83.51 (4)	C10—C11—H11E	109.5
N1—Cu—Cu^i^	176.88 (4)	H11A—C11—H11E	56.3
C1—N1—C7	117.39 (16)	H11B—C11—H11E	141.1
C1—N1—Cu	119.81 (12)	H11C—C11—H11E	56.3
C7—N1—Cu	122.68 (12)	H11D—C11—H11E	109.5
C10—O1—Cu	124.66 (12)	C10—C11—H11F	109.5
C10—O2—Cu^i^	122.03 (12)	H11A—C11—H11F	56.3
C12—O3—Cu	123.63 (12)	H11B—C11—H11F	56.3
C12—O4—Cu^i^	123.05 (12)	H11C—C11—H11F	141.1
N1—C1—C8	124.11 (17)	H11D—C11—H11F	109.5
N1—C1—H1	117.9	H11E—C11—H11F	109.5
C8—C1—H1	117.9	O4—C12—O3	125.49 (17)
C3—C2—C8	119.9 (2)	O4—C12—C13	117.46 (18)
C3—C2—H2	120.0	O3—C12—C13	117.04 (18)
C8—C2—H2	120.0	C12—C13—H13A	109.5
C2—C3—C4	120.6 (2)	C12—C13—H13B	109.5
C2—C3—H3	119.7	H13A—C13—H13B	109.5
C4—C3—H3	119.7	C12—C13—H13C	109.5
C5—C4—C3	121.1 (2)	H13A—C13—H13C	109.5
C5—C4—H4	119.5	H13B—C13—H13C	109.5
C3—C4—H4	119.5	C12—C13—H13D	109.5
C4—C5—C9	120.0 (2)	H13A—C13—H13D	141.1
C4—C5—H5	120.0	H13B—C13—H13D	56.3
C9—C5—H5	120.0	H13C—C13—H13D	56.3
C7—C6—C9	119.90 (18)	C12—C13—H13E	109.5
C7—C6—H6	120.1	H13A—C13—H13E	56.3
C9—C6—H6	120.1	H13B—C13—H13E	141.1
C6—C7—N1	123.52 (17)	H13C—C13—H13E	56.3
C6—C7—H7	118.2	H13D—C13—H13E	109.5
N1—C7—H7	118.2	C12—C13—H13F	109.5
C1—C8—C9	117.99 (16)	H13A—C13—H13F	56.3
C1—C8—C2	122.54 (17)	H13B—C13—H13F	56.3
C9—C8—C2	119.46 (17)	H13C—C13—H13F	141.1
C8—C9—C5	118.87 (18)	H13D—C13—H13F	109.5
C8—C9—C6	117.09 (17)	H13E—C13—H13F	109.5
C5—C9—C6	124.04 (19)		

Symmetry codes: (i) −*x*+1, −*y*+1, −*z*+1.
